# Resting-State Functional Connectivity Characteristics of Resilience to Traumatic Stress in Dutch Police Officers

**DOI:** 10.3389/fnbeh.2022.919327

**Published:** 2022-07-27

**Authors:** Santoucha N. W. Setroikromo, Steven J. A. van der Werff, Annika S. Smit, Eric Vermetten, Nic J. A. Van Der Wee

**Affiliations:** ^1^Department of Psychiatry, Leiden University Medical Center, Leiden, Netherlands; ^2^Leiden Institute for Brain and Cognition, Leiden, Netherlands; ^3^Rivierduinen, Institute for Mental Health Care, Leiden, Netherlands; ^4^Leids Universitair Behandel- en Expertise Centrum, Leiden, Netherlands; ^5^Dutch Police Academy, Apeldoorn, Netherlands

**Keywords:** MRI, stress, resilience, trauma, resting-state, police officers, functional connectivity

## Abstract

**Background:**

Insights into the neurobiological basis of resilience can have important implications for the prevention and treatment of stress-related disorders, especially in populations that are subjected to high-stress environments. Evaluating large-scale resting-state networks (RSNs) can provide information regarding resilient specific brain function which may be useful in understanding resilience. This study aimed to explore functional connectivity patterns specific for (high) resilience in Dutch policemen after exposure to multiple work-related traumatic events. We investigated resting-state functional connectivity (RSFC) of the salience network (SN), limbic network, and the default-mode network (DMN).

**Methods:**

Resting-state functional MRI scans were obtained from trauma-exposed executive personnel of the Dutch police force and non-trauma-exposed recruits from the police academy. Participants were divided into three groups: a resilient group (*n* = 31; trauma exposure; no psychopathology), a vulnerable group (*n* = 32; trauma exposure, psychopathology), and a control group (*n* = 19; no trauma exposure, no psychopathology). RSFC of the three networks of interest was compared between these groups, using an independent component analysis and a dual regression approach.

**Results:**

We found decreased resilience-specific positive RSFC of the salience network with several prefrontal regions. The DMN and limbic network RFSC did not show resilience-specific patterns.

**Conclusion:**

This study shows a differential RSFC specific for resilient police officers. This differential RSFC may be related to a greater capacity for internal-focused thought and interoceptive awareness, allowing more effective higher-order responses to stress in highly resilient individuals.

## Introduction

The way individuals react and respond to extreme stress and adversity differs significantly. Some individuals may develop psychiatric disorders, such as anxiety or mood disorders, whereas others are considered more resilient, recovering from stressful experiences displaying no or only minimal symptoms of psychological distress and without functional impairment. Resilience can be conceptualized as “*a dynamic developmental process encompassing the attainment of positive adaptation within the context of significant threat, severe adversity, or trauma” (Cicchetti, 2010)*.

First responders such as police officers are more likely to experience traumatic events through the nature of their work. However, at the same time, a lower incidence of psychopathology is typically reported in this population (Skogstad et al., 2013; van der Velden et al., 2013). This makes first responders an interesting group to study mechanisms involved in resilience to traumatic stress.

Psychological factors involved in resilience have been studied extensively in first responders, military, and other populations and were found to include among other positive trauma-related reappraisal, emotional flexibility, social problem-solving, coping strategies, and personality traits (Luthar et al., 2000; Franklin et al., 2012). Studies have found a “resilient personality” to consist of a pattern of low neuroticism, an above average extroversion, openness, agreeableness, and conscientiousness (Campbell-Sills et al., 2006; Berry et al., 2007; Hjemdal et al., 2012). In addition, resilient individuals are generally optimistic and are characterized by high positive emotionality, possessing a specific explanatory style with coping strategies including positive reappraisal and acceptance (Southwick et al., 2005).

In contrast to the amount of data on the role of psychological factors, data on the neurobiology of resilience are still limited. Studies suggest the involvement of various neurotransmitter and hormone systems, genetic and epigenetic factors, and specific neurocircuitry (see review: Osorio et al., 2017). A previous review suggests that the neural circuitry of resilience includes among other key brain structures involved in emotion and stress regulation, e.g., the limbic network, rendering increased emotion regulation capacities in resilient individuals (van der Werff et al., 2013b). These key structures include the amygdala, insula, hypothalamus, hippocampus, and cortical structures such as the medial prefrontal cortex (mPFC) and the anterior cingulate cortex (ACC).

Neuroimaging has become an increasingly important tool to study neural correlates of behavior *in vivo*. Resting-state functional magnetic resonance imaging (RS-fMRI) relies on intrinsic brain activity, i.e., brain activity that is not induced by an external stimulus. When an individual is at rest, spontaneous low-frequency fluctuations in the blood oxygenation level-dependent (BOLD) response have been shown to temporally correlate between regions in large-scale functional brain networks also known as resting-state networks (RSNs) (Beckmann et al., 2005; Fox and Raichle, 2007; Smith et al., 2009). Thus, evaluating RSNs can provide information regarding inherent brain function that may help to identify network key to a resilient profile, which may be useful in further unraveling the neurobiology of resilience. Altered functional connectivity of the default-mode network (DMN) and the salience network (SN) have been linked to resilience as well as to stress-related psychopathology. The DMN is thought to be involved in self- and environmental referential mental activity, memory formation, and spontaneous thought. The DMN comprises the MPFC, the posterior cingulate cortex (PCC), precuneus, and the left and right inferior parietal lobules/angular gyrus (Smith et al., 2009; Mantini and Vanduffel, 2013). Altered DMN functional connectivity has been linked to the impact of early life stress (Whitfield-Gabrieli and Ford, 2012; Philip et al., 2013), as well as post-traumatic stress disorder (PTSD) (Lanius et al., 2010; Daniels et al., 2011). The SN is thought to play an important role in the integration of sensory information including the implementation of goal-directed tasks and consists of the dorsal anterior cingulate cortex (dACC) and bilateral insulae (Seeley et al., 2007). Altered SN functional connectivity has been linked to PTSD in veterans (Sripada et al., 2012) and resilience to childhood maltreatment (van der Werff et al., 2013a). In addition, SN modulation has been linked to DMN and central executive network (CEN) functioning, making proper SN functioning necessary for effective cognitive control (Bonnelle et al., 2012).

In this study, we aim to identify resting-state functional connectivity (RSFC) patterns specific for resilience in a unique sample of Dutch police officers. Previous studies mainly focused on the differences between resilient vs. non-resilient individuals which limits conclusions on resilient specific correlates (for an overview see: Koch et al., 2016). We therefore decided to compare resilient individuals (RES = trauma-exposed police officers without psychopathology) with two groups: a vulnerable group (VUL = trauma-exposed police officers with psychopathology) and a control group (CON = non-trauma-exposed police officers without psychopathology). We specifically included a wide variety of different trauma-related psychopathologies within the vulnerable group to be able to study resilience to trauma exposure in general rather than factors that protect against specific trauma-related psychopathologies.

In addition, previous studies that compare RSFC between resilient vs. non-resilient individuals has focused on connectivity patterns of circumscribed brain regions (e.g., seed-based analysis) instead of RSFC of functional systems that are linked to support core perceptual and cognitive processes (RSNs). We therefore chose to explore functional connectivity within large-scale RSNs as well as between large-scale RSNs and the rest of the brain.

Based on the existing literature, we focused on three RSNs comprising the limbic network, the DMN, and the SN. Based on the previous literature, we hypothesized that we would detect resilience-specific patterns in functional connectivity within the limbic, salience, and DMNs. In addition, we hypothesized that these differences in functional connectivity were correlated with resilient coping strategies.

## Materials and Methods

### Participants

Trauma-exposed executive personnel of the Dutch police force and non-trauma-exposed recruits from the police academy were recruited through advertisements on the intranet of the Dutch police. About 149 participants signed up for the study and were subsequently screened for exclusion criteria. Participants were enrolled in the study if they did not meet the following exclusion criteria: (i) MRI contraindications, (ii) a history of neurological or other medical illness, (iii) the use of psychotropic medication other than stable use of selective serotonin reuptake inhibitors (SSRI) or infrequent benzodiazepines use (i.e., equivalent to 2 doses of 10 mg of oxazepam 3 times per week as a maximum and refrain from use 48 h before scanning), (iv) a history of childhood maltreatment (i.e., < 18 years) was included as an exclusion criteria due to evidence of specific brain structural and functional characteristics related to childhood maltreatment as well as the relation of the experience of childhood maltreatment and the development of stress-related psychiatric disorders in later life, (v) a history of psychopathology with onset before work-related traumatic events, (vi) left-handedness, (vii) insufficient knowledge of the Dutch language, and (viii) smoking > 5 cigarettes a day on average. After screening, 86 participants were invited to the hospital for data acquisition. A total of four participants were excluded from the study after quality checking the MRI data, due to motion-related noise. Therefore, the total sample size of this study was 82 participants. The participants were divided into three groups based on two criteria: (1). work-related trauma exposure as measured by the Police Life Events Schedule (PLES) and (2). Meeting criteria of one or more DSM-IV diagnoses either current or past according to the Mini-International Neuropsychiatric Interview (MINI). The RES group (*N* = 31) included individuals who experienced multiple work-related traumatic events, without the presence of a current or past DSM-IV diagnosis. The VUL group (*N* = 32) included individuals who experienced multiple work-related traumatic events, with the presence of one or more current or past DSM-IV diagnoses. The CON group (*N* = 19) included individuals recruited from the police academy without exposure to traumatic experiences and without the presence of a current or past DSM-IV diagnosis. The control group was recruited from the police academy to keep the groups as homogeneous as possible with respect to personality characteristics, which was deemed as more important than matching on age.

After explanation of the procedure, all participants signed informed consent. The study protocol was approved by the medical ethical committee of the Leiden University Medical Center under protocol number NL40761.058.12. The study was designed and conducted in accordance with the principles of the Declaration of Helsinki.

### Behavioral Assessment

The assessment of past and current psychiatric disorders was determined using the MINI. The MINI is an interview used to assess the presence of the most common Axis 1 psychiatric disorders according to DSM-IV and ICD-10 criteria (van Vliet and de Beurs, 2007). The Montgomery-Asberg Depression Rating Scale (MADRS) is a 10-item diagnostic questionnaire used to measure the severity of depressive episodes in patients with mood disorders (Montgomery and Asberg, 1979). The Inventory of Depression Symptomatology (IDS) is a self-report questionnaire with 28 items, measuring the presence and severity of symptoms of depression (Rush et al., 1996). Internal consistencies (Cronbach’s alpha) range from 0.76 to 0.94. The Beck’s Anxiety Inventory (BAI) was administered to assess the severity of anxiety symptoms (Beck et al., 1988). The BAI consists of twenty-one questions regarding how the subject has been feeling in the last week, expressed as common symptoms of anxiety. Cronbach’s alpha for the Dutch version of this inventory was found to be 0.82. Furthermore, the Harvard Trauma Questionnaire (HTQ) was assessed to evaluate the variety of trauma and the severity of the corresponding emotions. This questionnaire consists of 30 items that can be scored from 1 to 4 points with a Cronbach’s alpha of 0.95 (Mollica et al., 1992). In addition, the degree of exposure to work-related life events was evaluated using the PLES. The PLES (Cronbach’s alpha = 0.87) is a 37-item measure of the type and number of traumatic incidents by police officers and the degree to which they felt threatened, anxious, and helpless at each of the incidents (Carlier et al., 1997). The traumatic incidents listed within the PLES can be considered criterium A for PTSD according to DSM-V guidelines and include but are not limited to threatening situations with various weapons, as well as exposure to victims of crimes or accidents resulting in death or severe bodily injury. There are separate items depending whether the victim is a child, an adult, or a colleague.

The cognitive emotion regulation questionnaire (CERQ) (Garnefski et al., 2001) was assessed to determine individuals cognitive coping strategies, which is defined as “an individual’s thoughts after having experienced a negative event.” The nine cognitive emotion regulation strategies are as follows: (i) self-blame, (ii) other-blame, (iii) rumination, (iv) catastrophizing, (v) putting into perspective, (vi) positive refocusing, (vii) positive reappraisal, (viii) acceptance, and (ix) planning. The first four strategies are considered maladaptive, and the latter five adaptive. The Connor-Davidson Resilience Scale (CD-RISC) (Connor and Davidson, 2003), a self-report questionnaire, was used to assess individual’s resilience level. The Connor-Davidson Resilience Scale (CD-RISC) comprises of 25 items, each rated on a 5-point scale (0–4), with higher scores reflecting greater resilience. The content of the scale features is among others: developing strategy with a clear goal or aim, action orientation, strong self- esteem/confidence, adaptability when coping with change, social problem-solving skills, humor in the face of stress, strengthening effect of stress, taking on responsibilities for dealing with stress, secure/stable affectional bonds, and previous experiences of success and achievement. The internal consistency for the full scale is Cronbach’s alpha = 0.89.

### Imaging Procedure

Scanning was performed on a Philips 3T MRI system (Philips Healthcare, Best, the Netherlands; software version 3.2.1), using a 32-channel head coil.

The following parameters were used to obtain a high resolution 3D T1-weighted image: repetition time = 9.8 ms, echo time = 4.6 ms, matrix size 256 × 256, voxel size 1.17 mm^3^ × 1.17 mm^3^ × 1.2 mm^3^, 140 slices, with a scan duration of 4:56 min.

RS-fMRI scans were acquired using T2*-weighted gradient-echo echo-planar imaging with the following scan parameters: 200 whole-brain volumes; repetition time (TR) = 2,200 ms, echo time (TE) = 30 ms, flip angle = 80°, 38 slices, matrix size = 80 × 80, voxel size = 2.75 mm^3^ × 2.75 mm^3^ × 2.75 mm^3^, with a scan duration of 7:28 min. All subjects were asked to close their eyes while staying awake and to lie as still as possible.

To facilitate registration of the functional image to standard space, a high-resolution T2*-weighted gradient-echo echo-planar imaging scan was required, with the following scan parameters TR = 2,200 ms, TE = 30 ms, flip angle = 80°, 84 axial slices, matrix size = 112 × 112, voxel size = 1.96 mm^3^ × 1.96 mm^3^ × 2 mm^3^, no slice gap, scan duration = 46.2 s).

### MRI Data Processing

All analyses were performed using FMRIB Software Library (FSL) (Smith et al., 2004), version 5.0.10. Preprocessing was carried out as described in the study of Pruim et al. (Pruim et al. (2015a,b)), in FSL’s FMRI Expert Analysis Tool (FEAT), version 6.00. Thereafter, head motion correction was performed using MCFLIRT (subject movement > 3 mm in any direction, resulted in exclusion of the data from further analysis; *N* = 4 participants were excluded due to excessive head motion) (Jenkinson et al., 2002), followed by non-brain removal, and spatial smoothing with a Gaussian kernel of 6 mm^3^ × 6 mm^3^ × 6 mm^3^ FWHM. The preprocessed RS images were registered into the corresponding brain extracted high-resolution T2*-weighted EPI image. The high-resolution T2*-weighted EPI image was then registered to the corresponding T1-weighted images and the T1-weighted images were registered to the MNI 152 standard space (2 mm isotropic). Registration into standard space was done after Automatic Removal Of Motion Artifacts based on independent component analysis (ICA-AROMA version 3.0-beta). ICA-AROMA automatically identifies and subsequently removes data-driven-derived components that represent motion-related artifacts, while preserving signal of interest. High pass temporal filtering (0.01 Hz) was done after denoising the fMRI data with ICA-AROMA. Thereafter, nuisance regression was performed, removing white matter (WM) and cerebrospinal fluid (CSF) signal.

### Analysis of Resting-State Functional Connectivity

**FIGURE 1:**
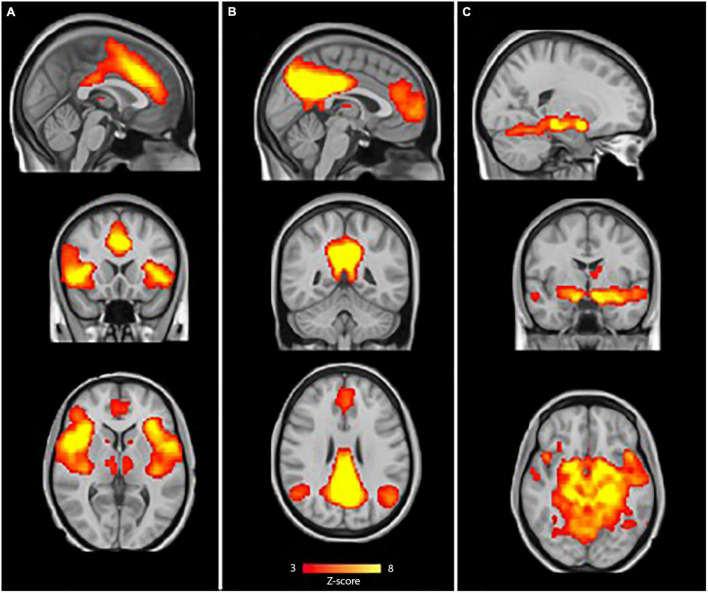
Resting-state networks of interest. The sample-specific selected components for: **(A)** Salience network (SN), **(B)** default-mode network (DMN) and **(C)**. Limbic network. The color bar depicts Z-scores and runs from red (*Z* = 3) to yellow (*Z* = 8).

FMRIB Software Library’s Multivariate Exploratory Linear Optimized Decomposition into Independent Components (MELODIC) tool was used to temporally concatenate all preprocessed data across subjects to create a single 4D dataset, which in turn was decomposed into 20 independent component analysis (ICA) components. These ICA components were visually inspected by two independent researchers and were selected by comparing the components with previously defined maps (Beckmann and Smith, 2004), resulting in the selection of three resting-state network of interest (Figure 1): the SN (A), the DMN (B), and the limbic network (C). Hereafter, dual regression was used to the set of spatial maps from the group-average analysis to generate subject-specific versions of the spatial maps and associated time series. The groups were then compared using a general linear model (GLM) including gender as confound regressor. To control for possible structural abnormalities and misregistration that could confound the differences in functional connectivity, gray matter values of each subject were included as a voxel-wise confound regressor in the GLM. Group differences were tested using permutation-based (5,000 permutations) non-parametric testing in a quadratic design. To control for family-wise error, threshold-free cluster enhancement (TFCE; Smith and Nichols, 2009) was applied and the threshold for significance was set on *p* < 0.05.

Correlation analyses for the difference in RSFC with CD-RISC as well as the subscales of the CERQ were performed with the use of Pearson’s r or with Kendall’s tau, when the data violated assumptions for parametric tests. All correlations were controlled for multiple comparisons using Bonferroni correction controlling for the number of (sub) scales tested, resulting in an adjusted *p*-value of 0.005.

## Results

Diagnoses in the VUL group consisted of one or more of the following: major depressive disorder (*N* = 27), anxiety disorder (*N* = 17), obsessive compulsive disorder (*N* = 2), posttraumatic stress disorder (*N* = 14), and substance abuse (*N* = 8). Furthermore, medication use in the VUL group was limited to stable use of either a SSRI (*N* = 7) or a non-selective SSRI (*N* = 1). Characteristics and between group statistics of the study population are reported in Table 1. There was a significant difference in age (*p* < 0.001) between the RES and CON group, due to younger cadets in the CON group. This difference in age was not found between the RES and VUL group (*p* = 0.277). Furthermore, there was a significant difference in gender ratio, with a higher male/female ratio in the RES group when compared to the CON group (*p* = 0.033).

**TABLE 1:** Characteristics of the study population.

	RES		VUL		CON		RES Vs. VUL	RES Vs. CON
	N		N		N		*P*-value	*P*-value
N	31		32		19			
Females/males	10/21		8/24		12/7		0.524^a^	**0.033*^a^***

	**Mean**	***SD***	**Mean**	***SD***	**Mean**	***SD***	***P*-value (Z or t-stat)**	***P*-value (Z or t-stat**)

Age	40.68	11.67	43.75	11.00	25.32	4.61	0.277^b^ (*Z* = 1.09)	**<0.001^b^ (*Z* = 4.70)**
IDS	36.39	6.82	43.94	12.65	32.58	5.32	**0.013^b^ (*Z* = 2.48)**	**0.017^b^ (*Z* = 2.40)**
BAI	24.00	2.73	26.31	6.56	23.94	3.00	0.183^b^ (*Z* = 1.33)	0.841^b^ (*Z* = 0.20)
MADRS	1.61	2.31	5.19	7.64	0.26	0.73	0.168^b^ (*Z* = 1.38)	**0.006^b^ (*Z* = 2.78)**
CD-RISC	98.23	11.92	92.25	14.44	103.89	9.57	0.079^c^ (*t* = 1.79)	0.086^c^ (*t* = 1.75)
HTQ	34.84	5.05	43.91	14.93	33.68	5.52	**0.010^b^ (*Z* = 2.59)**	0.159^b^ (*Z* = 1.41)
PLES (with outlier)	166.61	144.65	330.31	621.26	27.53	53.60	0.564^b^ (*Z* = 0.577)	**<0.001^b^ (*Z* = 4.61)**
PLES (outlier omission)	166.61	144.65	231.68	277.73	27.53	53.60	0.709^b^ (*Z* = 0.37)	**<0.001^b^ (*Z* = 4.61)**
CERQ: Self-blame	7.55	2.68	8.59	3.32	7.95	2.32	0.211^b^ (*Z* = 1.25)	0.449^b^ (*Z* = 0.76)
CERQ: Other-blame	5.74	1.79	7.16	2.58	5.42	1.71	**0.026^b^ (*Z* = 2.23)**	0.575^b^ (*Z* = 0.56)
CERQ: Rumination	10.06	3.82	12.06	6.82	8.79	3.39	0.183^b^ (*Z* = 1.33)	0.248^b^ (*Z* = 1.12)
CERQ: Catastrophizing	4.87	1.50	6.34	3.01	4.74	1.19	**0.003^b^ (*Z* = 2.95)**	0.811^b^ (*Z* = 0.24)
CERQ: Putting into perspective	11.71	4.02	11.31	3.42	13.05	3.54	0.674^c^ (*t* = 0.423)	0.236^c^ (*t* = 1.20)
CERQ: Positive refocusing	11.45	4.22	11.41	3.39	11.74	3.66	0.963^c^ (*t* = 0.047)	0.809^c^ (*t* = 0.24)
CERQ: Positive reappraisal	14.55	3.41	14.16	3.81	15.37	3.44	0.934^b^ (*Z* = 0.083)	0.387^b^ (*Z* = 0.86)
CERQ: Acceptance	10.42	2.84	12.44	3.14	12.68	3.30	**0.011^b^ (*Z* = 2.56)**	**0.018^b^ (*Z* = 2.36)**
CERQ: Planning	13.58	3.62	13.94	3.15	14.26	2.75	0.678^c^ (*t* = 0.42)	0.484^c^ (*t* = 0.71)

*IDS, Inventory of depression symptomatology; BAI, Becks Anxiety Inventory; MADRS, Montgomery-Asberg Depression Rating Scale; CD-RISC, Connor-Davidson Resilience Scale; HTQ, Harvard Trauma Questionnaire; PLES, Police Life Events Schedule; CERQ, Cognitive Emotion Regulation Questionnaire. ^a^Chi-square; ^b^Mann Whitney U; ^c^independent sample t–test.*

*Values considered to be statistically significant are displayed in Bold.*

The number of experienced work-related life events, measured with the PLES, was significantly higher in the RES group compared to the CON group (*p* < 0.001), but not compared to the VUL group (*p* = 0.564). One outlier was present in the VUL group, reporting 3,388 work-related life events by one individual, for details, refer to van der Werff et al. (2017) (24). After omission of this outlier, the difference between mean PLES scores of the RES and VUL group remained non-significant (*p* = 0.709). The trauma-related severity, measured with the HTQ, was significantly lower in the RES group in comparison with the VUL group (*p* = 0.010), but not in comparison with the CON group (*p* = 0.159). Depression scores, measured with the IDS, were significantly lower (*p* = 0.013) in the RES compared to the VUL group, but higher (*p* = 0.017) when compared to the CON group. In addition, MADRS scores were significantly higher in the RES compared to the CON group (*p* = 0.006).

With regard to the cognitive emotion regulation strategies, the RES group had significant lower scores on the subscale acceptance compared to both the CON (*p* = 0.018) and the VUL groups (*p* = 0.011). In addition, the RES group scored significantly lower on the subscales other-blame (*p* = 0.026) and catastrophizing (*p* = 0.003) compared to the VUL, but not the CON group.

No significant differences between the RES and the VUL or CON group were found in BAI score or CD-RISC score.

### Resting-State Functional Connectivity

**FIGURE 2:**
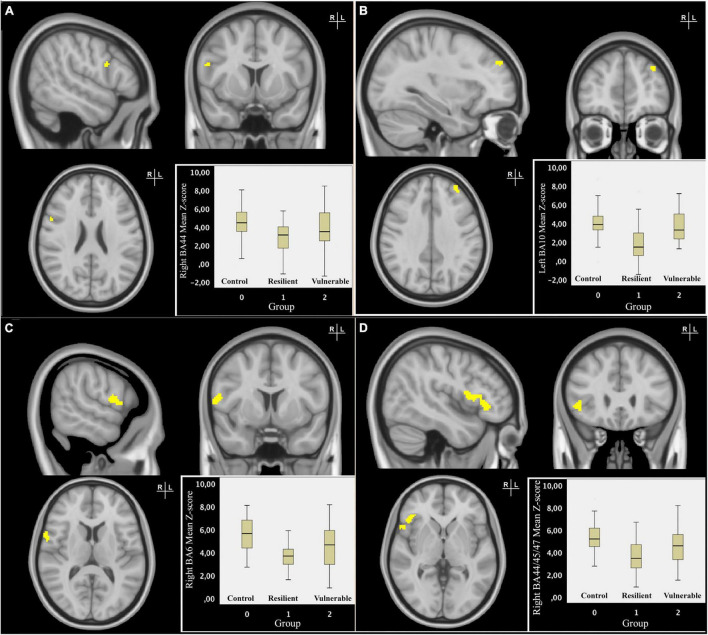
Resilience-specific resting-state functional connectivity (RSFC) of the salience network (SN). Coronal, sagittal and transversal slices of the MNI-152 0.5 mm standard brain. The RES group showed significant differential RSFC of the right BA44 **(A)** and left BA10 **(B)** with the salience network compared to the VUL and CON group. The significant clusters were threshold at *p* < 0.05 and subsequently binarized in a mask. The boxplot displays mean individual z-scores of these clusters, depicted separately for each group. In radiological convention, the right side of the image corresponds to the left side of the brain. Coronal, sagittal, and transversal slices of the MNI-152 0.5 mm standard brain. The RES group showed significant differential RSFC of the right BA6 **(C)** and right BA44/45/47 **(D)** with the salience network compared to the VUL and CON groups. The significant clusters were threshold at *p* < 0.05 and subsequently binarized in a mask. The boxplot displays mean individual z-scores of these clusters, depicted separately for each group. In radiological convention, the right side of the image corresponds to the left side of the brain.

The quadratic design identified four clusters showing a significant difference in RSFC with the SN between the RES group and the VUL group, and between the RES and CON group after FDR correction (Figure 2). The mean individual z-scores for these clusters were extracted from the subject-specific z-maps of the component representing the SN connectivity, which is depicted in the boxplot per (Figures 2A–D). When comparing the RES with VUL group as well as with the CON group, the mean individual z-scores showed a decrease in positive connectivity of the SN with: the right inferior frontal gyrus (BA44), right precentral gyrus/supplementary (pre)motor area (SMA) (BA 6), the right ventrolateral (BA44,45,47), and left lateral (BA10) parts of the PFC (Table 2).

**TABLE 2:** Characteristics of resilience-specific clusters of the salience network (SN).

Region	Brodmann area	Voxel size	Peak of activation (voxel) X, Y, Z	*P*-value
Inferior frontal gyrus (R)	44	10	68, 18, 48	0.025
Lateral prefrontal cortex (L)	10	29	87, 62, 53	0.019
Precentral gyrus/supplementary (pre)motor area (R)	6	118	67, 15, 42	0.025
Ventrolateral prefrontal cortex (R)	44, 45, 47	269	66, 21, 38	0.046

*The size of the clusters is given using a threshold of 0.05.L, left; R, Right.*

There was no association between coping styles (measured with CERQ and CD-RISC) and the mean individual z-scores from the significant clusters in the RES group. Furthermore, between group comparisons of the DMN and the limbic network did not show significant difference in RSFC.

## Discussion

In this study, we explored DMN, SN, and limbic network resting-state connectivity in a sample consisting of resilient Dutch police officers (i.e., trauma-exposed police officers who did not developed psychopathology), trauma-exposed police officers who developed psychopathology, and a non-exposed control group consisting of cadets from the Police Academy. We hypothesized that RES police officers would be characterized by specific RSFC patterns of the limbic, SN, and DMN networks. In addition, we hypothesized that differences in functional connectivity would be correlated with psychometric scores for resilience. In the RES group, we found a differential (i.e., decreased positive) connectivity of the SN with the inferior frontal gyrus (BA44), the precentral gyrus/supplementary (pre)motor area (SMA) (BA 6), ventrolateral (BA44,45,47), and lateral (BA10) parts of the PFC. It is important to note that we did not control for multiple comparisons across networks. All findings should therefore be considered explorative and should be interpreted with care.

There were no correlations between psychometric resilient characteristics and this resilience-specific RSFC pattern of the SN within the resilient group. Finally, we found no resilience-specific connectivity patterns for the DMN or limbic network.

The regions that showed a resilience-specific RSFC pattern with the SN typically have been associated with cognitive assessment of environmental stimuli and regulation of behavior and emotions (Farrow et al., 2012). For instance, lateral prefrontal regions have been implicated in cognitive control relevant to emotion, such as establishing increased attention control over expected threat-related distractors (Bishop et al., 2004). Of particular relevance, the lateral orbitofrontal cortex/ventrolateral PFC is thought to play a key role in the balance between the SN and CEN activities (Sridharan et al., 2008), with extensive interconnections to the amygdala as well as to the medial and lateral PFC (Dixon et al., 2017). In addition, the right ventrolateral prefrontal cortex is thought to be involved in judgment of relative salience. This judgment of the salience signal is then carried on to the premotor areas to implement response inhibition (Walther et al., 2011).

The SN plays a major role in the interactions between brain networks and one of its key roles, apart from the detection of salient internally or externally sensory information, is the integration of top-down appraisal and bottom-up information (Seeley et al., 2007) and the subsequent switching between activity of the DMN and CEN. Furthermore, it is thought that the SN initiates the CEN to respond to salient information for attentional shifts and to control execution (Menon and Uddin, 2010). The CEN mainly includes the dorsal lateral prefrontal cortex and the lateral parietal cortex, dorsomedial frontal/pre-SMA, and ventrolateral prefrontal cortex (Seeley et al., 2007). During rest, the SN and CEN are deactivated, which is thought to enable internally focused thought, interoceptive awareness, and processing (Sridharan et al., 2008). Taken together, the present result of a less positive RSFC between elements of the CEN (i.e., the ventrolateral/dorsolateral parts of the PFC) with the SN in both group comparisons (i.e., resilient vs. psychopathology and resilient vs. controls) suggests that this connectivity pattern is specific for resilience.

In line with the recent views on the role of the SN and the DMN, our findings may be interpreted as suggesting that resilient police officers have a greater capacity for internal-focused thought and interoceptive awareness, which would potentially enable higher-order processing of emotional information in the sequelae of traumatic events. Of interest, several studies in military personnel and top athletes have examined the effects of mindfulness based training (MFBT) on performance during very stressful situations and have also identified brain correlates of MFBT during task fMRI. The results showed that MFBT led to better performance in very stressful situations and to the changes in insula reactivity (a key region in the SN), and, in line with our findings, changes in the connectivity of the insula with prefrontal regions, notably the ACC. These changes were interpreted as allowing more effective higher-order responses to stress through more efficient interoceptive processing (Johnson et al., 2014; Haase et al., 2016). A more recent study also found increased within SN connectivity in an adolescent trauma-exposed control group further implying the role of the SN in resilience to trauma exposure (Sheynin et al., 2020).

Interestingly, this interoceptive processing capacity seems hampered in patients with PTSD as shown by a recent meta-analysis and systematic review of resting-state studies in PTSD (Koch et al., 2016) reporting that enhanced SN connectivity is associated with increased salience processing and hypervigilance in patients with PTSD. In addition, in another recent review (Akiki et al., 2017), PTSD was concluded to be associated with an overactive and hyperconnected SN during rest, suggesting hypervigilance in these patients at the cost of reduced awareness of internal-focused thoughts, interoceptive awareness, and autobiographical memory.

We did not find an association between coping styles, as measured with CERQ and CD-RISC, and strength of resilience-specific RSFC in the RES group. This could be due to our sample sizes and/or to the fact that these types of psychometric scales are often very heterogeneous in nature and hence do not “map” precisely on (resting-state) brain characteristics.

Contrary to our expectations, the DMN RSFC and limbic RFSC did not show resilience-specific patterns, perhaps because we examined three selected groups with similar baseline trait resilience levels. Finally, it is well possible that differences in functional activity within the limbic network might only be expressed when demands on cognition are high (i.e., during a stress paradigm or tasks).

To the best of our knowledge, this study was the first to investigate resilience-specific RSFC correlates in police officers and used a design with three groups that allows to identify resilience-specific correlates. Our study has also some potential limitations. First, our cross-sectional study design did not allow us to investigate causality, i.e., the role of differential RSFC in the development of resilience or as an acquired post-trauma characteristic. Longitudinal studies are needed to investigate this issue. Second, regarding our study sample, our findings should probably be regarded as specific to “highly resilient” populations, as police officers are, based on the stringent selection, and training, already more resilient to stress than the general population. Remarkably, depression symptomatology scores as measured with the MADRS differed between our RES group and the control group, suggesting a higher level of depressive symptoms within the RES group. However, both groups scored extremely low (mean MADRS score of the RES group = 1.61 and mean score of the control group = 0.26), suggesting an absence of depressive symptomatology in both groups. Differentiating between *“trait resilience levels”* can be addressed in future studies by adding a fourth, non-trauma-exposed healthy control group from the general population. In addition, we did not add age as a regressor due to the characteristics of the control group, which consists of young non-trauma-exposed police cadets. Furthermore, we did not correct for the number of networks investigated in this study, giving its explorative nature. In addition, our sample size could be considered a limitation. Finally, it remains unknown how the investigated networks relate to their function during tasks and how cognitive control mechanisms are mediated during active cognitive processing. Therefore, future studies should also incorporate task-based fMRI paradigms to investigate connectivity and coupling of brain networks under stress and during emotion regulation tasks or trauma scripted paradigms.

In summary, our explorative study shows a differential RSFC between the SN and key structures of the CEN specific for resilient police officers. This differential RSFC may be related to a greater capacity for internal-focused thought and interoceptive awareness, allowing more effective higher-order responses to stress in highly resilient individuals.

## Data Availability Statement

The data that support the findings of this study are available from the corresponding author, SW, S.J.A.van_der_Werff@lumc.nl, upon reasonable request. For reasons of privacy the data cannot be made publicly available.

## Ethics Statement

The studies involving human participants were reviewed and approved by Medisch Ethische Toetsingscommissie (METC) (https://www.metc-ldd.nl/). The patients/participants provided their written informed consent to participate in this study.

## Author Contributions

SW acquired the data. AS facilitated logistics surrounding participants. SW and SS organized the database and performed statistical analysis. SS wrote the first draft of the manuscript under supervision of SW and NV. All authors contributed to manuscript revision, read, and approved the submitted version and contributed to the conception and design of the study.

## Conflict of Interest

The authors declare that the research was conducted in the absence of any commercial or financial relationships that could be construed as a potential conflict of interest.

## Publisher’s Note

All claims expressed in this article are solely those of the authors and do not necessarily represent those of their affiliated organizations, or those of the publisher, the editors and the reviewers. Any product that may be evaluated in this article, or claim that may be made by its manufacturer, is not guaranteed or endorsed by the publisher.
